# GROWING UP WITH OPEN SCIENCE

**DOI:** 10.1093/geroni/igz038.087

**Published:** 2019-11-08

**Authors:** Gabrielle N Pfund

**Affiliations:** Washington University in St. Louis, St. Louis, Missouri, United States

## Abstract

While a novel concept for many, pre-registration is an outlet growing in prevalence that allows researchers to systematically work through future studies from start to finish by laying out research questions, relevant hypotheses, and study design. “Growing up” as a researcher with Open Science, I never felt that pre-registration limited me, my ability to think and ask questions, or any opportunities to explore new ideas with old data. Instead, it strengthened me as a researcher by giving me a methodical perspective to take when considering why I needed to ask a question and how I planned to answer it. In this talk, I will define what pre-registration is and discuss my experiences with it as an early career researcher. Furthermore, I will present different concerns and conceptions people have about it and expound upon the inherent benefits it provides to both critical thinking and study design.

